# Efficient Anomaly Detection for Smart Hospital IoT Systems

**DOI:** 10.3390/s21041026

**Published:** 2021-02-03

**Authors:** Abdel Mlak Said, Aymen Yahyaoui, Takoua Abdellatif

**Affiliations:** 1 SERCOM Lab, University of Carthage, Carthage 1054, Tunisia; takoua.abdellatif@ept.rnu.tn; 2 Military Academy of Fondouk Jedid, Nabeul 8012, Tunisia

**Keywords:** internet of things, smart hospitals, anomaly detection, intrusion detection, event detection, routing attacks, machine learning, RPL

## Abstract

In critical Internet of Things (IoT) application domains, such as the Defense Industry and Healthcare, false alerts have many negative effects, such as fear, disruption of emergency services, and waste of resources. Therefore, an alert must only be sent if triggered by a correct event. Nevertheless, IoT networks are exposed to intrusions, which affects event detection accuracy. In this paper, an Anomaly Detection System (ADS) is proposed in a smart hospital IoT system for detecting events of interest about patients’ health and environment and, at the same time, for network intrusions. Providing a single system for network infrastructure supervision and e-health monitoring has been shown to optimize resources and enforce the system reliability. Consequently, decisions regarding patients’ care and their environments’ adaptation are more accurate. The low latency is ensured, thanks to a deployment on the edge to allow for a processing close to data sources. The proposed ADS is implemented and evaluated while using Contiki Cooja simulator and the e-health event detection is based on a realistic data-set analysis. The results show a high detection accuracy for both e-health related events and IoT network intrusions.

## 1. Introduction

Recently, the deployment of the Internet of Things (IoT) has become highly recommended in many applications in different fields. IoT relies on sensors that collect data from the environment in order to ensure tasks, such as surveillance or monitoring for wide areas [1]. This capability gave birth to the notion of smart infrastructures, such as smart metering systems, smart city, and smart grid, etc. This paper focuses on the e-health field [2] and specifically on smart hospital infrastructures. In such infrastructures, sensors are collecting patients’ and their environment data and sending them to intermediate gateways. The gateways forward data to border routers while using routing protocols. Data analysis may take place at the border router, or data may be sent to the cloud servers for storage and advanced analysis.

**Figure 1:**
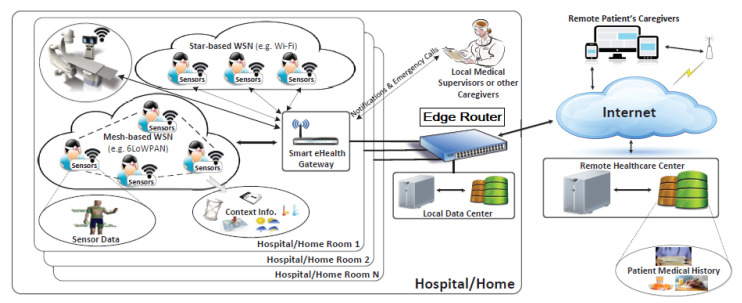
Smart Hospital infrastructure.

Smart hospital infrastructures, as shown in Figure 1 [3], have a wide range of resources that are essential in maintaining their operations, patients, employees, and the building safety, as demonstrated in the following [4,5]:

- Remote care assets:medical equipment for tele-monitoring and tele-diagnosis.
- Networked medical devices: wearable mobile devices (heartbeat bracelet, wireless temperature counters, glucose measuring devices, etc.) or equipment installed to collect health service related data.
- Networking equipment: standard equipment providing connectivity between different equipment (transmission medium, router, gateway, etc.).
- Data: may be related to patients or staff information as well as the equipment and environment information. It is considered to be the most critical asset to protect. It may be stored in local data centers as well as remote Healthcare centers.
- Building and facilities: the sensors are distributed in the hospital building and rooms to preserve the patient safety (temperature sensors in patient rooms and operating theatre and gas sensors in hospital kitchen are among the used sensors).

A common IoT architecture is used, and that can be considered for smart hospitals [5]. In such architecture, there are mainly three types of components:

- IoT nodes: composed of remote care assets, network medical devices, and different sensors and gateways. Sensors will send different types of data and information (patient and staff data, medical equipment status, etc.) to the nearest gateway [6].
- Edge router: an edge router or border router is a specialized router residing at the edge or boundary of a network. This node ensures the connectivity of its network with external ones: a wide area network or the Internet. The edge router uses an external border gateway protocol, which is used extensively over the Internet to provide connectivity with remote networks and cloud services [7]. The edge router may be used in analysis tasks and send notifications to appropriate users.
- Visualization module and database: this module is the terminal of the network containing all of the collected data from the different nodes of the network. Data are visualized to users based on their roles in order to ensure patient safety and improve the healthcare system. The visualization module and database may be located in the local network as well as in cloud servers.

The end nodes are, in general, limited in terms of computational resources, battery, and memory capacities. New protocols are proposed under the IoT paradigm in order to optimize energy consumption and computation. For instance, 6LoWPAN protocol (IPv6 over Low Power Wireless Private Area Network) was introduced to cope with IoT device growth. Also, RPL, the IPv6 Routing Protocol for Low-Power and Lossy Networks, is currently considered the primary routing protocol for the Internet of Things (IoT). Many e-health IoT applications are based on these protocols, since they are designed for constrained devices in recent IoT applications. Nevertheless, these protocols are exposed to a variety of attacks that badly affect the process of patients’ care and the whole infrastructure monitoring. According to a recent Nokia report [8], attacks on IoT devices are continuously increasing at an alarming rate. This is due to the use of automated tools exploiting their vulnerabilities. The report states that IoT devices now make up roughly 33% of infected devices. The percentage was 16% in 2019 (a increase of 100%). These statistics are generated from aggregated data from monitoring network traffic on more than 150 million devices globally. Researchers also claim that more than half of all IoT devices are vulnerable to medium- or high-severity attacks. For e-health systems, a study in [9] depicts that 89% of healthcare organizations experienced a data breach in the past two years. Additionally, in [10], it is estimated that the loss of data and related failures will cost healthcare companies nearly six trillion dollars in damages in the next three years when compared to three trillion in 2017.

### 1.1. Problem Statement and Assumptions

In smart hospital infrastructures, a huge amount of sensitive data is exchanged among nodes throughout radio interfaces. These data have to be protected for accurate event detection and decision making. For example, the privacy of patients must be ensured in order to prevent unauthorized identification and information theft. Moreover, the hospital building itself is sensitive since many patients are in difficult situations. Consequently, maintaining the stability of the building and protecting it from different abnormal event is a challenge itself. For example, a temperature increase or decrease can harm the patient in operating rooms. Anomalies are generally data that deviate for usual patterns. In the context of smart hospitals, there are two types of anomalies: e-health related data anomalies and IoT network anomalies. E-health related data anomalies are generated by events, such as fires (unusual temperature and humidity data), patient state change (unusual heart beat rates), etc. These anomalies are very critical and they must be detected in real-time with no false alerts, especially for hospital infrastructures. On the other side, IoT network anomalies are generally characterized by an unusual change in the network traffic and nodes behaviors. They are mainly generated by network intrusions and nodes failures. Therefore, such type of anomaly has become a high priority challenge for researchers to preserve data accuracy and avoid sending false alerts. For the sake of anomaly detection, the analysis task may take place at local network or at cloud servers. However, the information treated in smart hospital infrastructures is very critical and latency sensitive. In fact, sending data to cloud servers for analysis, and decision making may increase the risk of security attacks. Also, this may lead to extra delay for sending data which depends on bandwidth and processing capability offered. In the particular case of smart hospital infrastructure, resources are available in contradiction with hostile environments surveillance [11] where other solutions are required especially for energy optimization.

### 1.2. Contribution

This paper is an extension of a previous work [12] which focuses on network attacks in IoT, more precisely, on the rank attack intrusion detection for IoT. In this paper, a larger scope of anomalies in a common anomaly detection system (ADS) is considered: (1) an event detection module for e-health identifying patients’ abnormal health state and (2) an intrusion detection module for IoT network anomalies detection. Having both modules in a unified system provides more reliable anomaly detection. Compared to existing IoT systems deployed in hospitals, the originality of this work consists in providing a common management system for e-health data and IoT networks supervision while current solutions generally separate both kinds of systems. Indeed, deployed sensors and processing units dedicated to patients’ care are generally hosted in a different system that network monitors. As soon as network intrusion is detected, data from non-trusted sources and networks is carefully processed, which avoids false alerts or emergency notification on-time. For example, no automated decision is taken from non-trusted data processing and checking data from its sources is required.

More precisely, integrating ADS subsystems (for intrusion and for event detection) in a single system has the following advantages:

- Centralizing data access for accurate decisions: by being able to read data from each subsystems in a centralized way is crucial for efficient decision making. Indeed, a Decision Manager component is introduced to decide if an event detection has to be trusted or not according to the intrusion detection information. If an intrusion is detected, events coming from the hospital system are immediately considered non-trusted until the intrusion issue is fixed by the administrator. In this e-health context, this feature is mandatory in order to avoid the false alerts and the wrong actions taken on patients’ care.
- Reducing the management costs: by using a common management application instead of several, administrators spend less time introducing data to the system, deploying and updating functional processes and thus, reducing energy consumption and engineering cost. Indeed, using several systems equals the necessity of switching from one platform to another in order to perform specific business tasks. In this case, a communication broker is adopted to automatically communicate intrusion information between decision makers, administrators and third party entities.

ADS modules are deployed at the border router to detect three different types of network intrusions as well as events of interest related to patient and environment states. The choice of edge computing [13] was driven by the reliability of decisions made inside the hospital. Indeed, sensitive data may be altered while sent to the cloud. Furthermore, processing data close to the sources reduces significantly the decision latency and bandwidth usage. We note that there are no resource constraints for the border router in hospitals IoT systems unlike other IoT environments where edge computing can be blocked by limited resources at the edge. Meanwhile, the sensors, like patients’ wearable sensors and environment’s temperature sensors, are resource-constrained. From the processing side, SVM (Support Vector Machines) algorithm is used as detection technique for its high detection accuracy and optimized execution time [14,15]. Other algorithms could be used and deployed but finding the best algorithms is out of the scope of this work. The proposed solution is efficient since both targets are reached: (1) the accurate decision regarding patients’ health state thanks to the reliability feature, and (2) the low communication between sensors and decision makers thanks to the edge-based deployment and to the optimal SVM processing.

### 1.3. Research Methodology

This work proposes a system for integrating e-health monitoring and hospital network infrastructure supervision. After a study of existent e-health systems and different IoT systems, an IoT architecture is proposed to ensure an efficient anomaly detection solution. For data analysis and processing, SVM is chosen as the detection technique based on state of art research comparison works. It was tested with two kinds of data-sets: one contains e-health data and the second contains network infrastructure data. As a placement strategy, a centralized solution at the edge of the network is chosen to ensure low notification latency and to protect sensitive data. Then, to validate the proposed architecture and chosen algorithm, a prototype is implemented and tested with different scenarios about e-health event detection and network infrastructure failures or intrusions. For e-health event detection, two use cases for fire and heart attack detection were investigated. For network infrastructure intrusion detection, three types of attacks were simulated and the detection accuracy was tested. For performance evaluation and to measure the scalability of the proposed system, A wireless sensor network simulator tool is used (Contiki Cooja simulator). The experimental results show the efficiency of this system in terms of accuracy in anomaly detection and short latency from sensing to action decision.

### 1.4. Paper’s Structure

The rest of the paper is structured as follows. Section 2 presents background details about the main treated concepts. Section 3 presents works related to this research field. Section 4 demonstrates the anomaly detection scenarios. Section 5 details the proposed approach. Section 6 displays the main results and evaluation, and Section 7 concludes the paper and refers to its perspectives. At the end, Appendix A, describes the implementation of the border router.

## 2. Background

The background section presents the edge and cloud computing concepts. Furthermore, it presents the 6LoWPAN and RPL protocols. At the end, SVM algorithm that is adopted in our work is presented.

### 2.1. IoT Edge and Cloud Computing

Since different IoT nodes generate huge amount of data, and since data flow needs high speed computing, edge and cloud analysis are presented as two alternatives for analysis placement [16]. Edge computing is executed on nodes close to IoT sensors, typically on gateways. This is required when data collection, transformation and analysis must be achieved in minimal latency. Cloud computing helps organizations to store and analyze large amounts of data on scalable hosting machines and leverage other services to save costs. The choice of placement depends generally on application latency, sensitivity, availability of resources, analysis task complexity, network bandwidth and security. Recent works propose placement on both locations [17] for better resource utilization and optimization. In some IoT systems, sensors and gateways are deployed in harsh environments and can hardly conserve their energy for long periods. In such systems, edge-processing has to be minimal to save node energy. In smart hospitals, there is no energy issue at edge nodes. Consequently, the edge computing solution can be used efficiently in order to enable millions of IoT devices to form an enormous intelligent network that handle locally e-health and IoT network data. In particular, data aggregation and analysis can be performed at the edge. The cloud is mainly used for persistence. Furthermore, the communication edge-cloud has to be secure since critical patients’ data is transferred.

### 2.2. 6LoWPAN and RPL

With the enormous number of devices that are now connected to the Internet, a new solution is proposed: 6LoWPAN (Low-Power Wireless Personal Area Networks). It is a lightweight protocol including packet compression and other optimization mechanisms that define how to run IP version 6 (IPv6) over low data rate, low power and small footprint radio networks as typified by the IEEE 802.15.4 radio [18]. The key feature of 6LoWPAN is interoperability insurance [19]. Thus, this standard has quietly gained significant ground. It has the ability to operate with other wireless standards making it an ideal choice for many applications in IoT. Moreover, 6LoWPAN uses IPv6, and this alone has to set it aside from other protocols with a distinct advantage. With the world migrating towards IPv6 packet data, a system such 6LoWPAN offers many advantages for low power wireless sensor networks and other forms of low power wireless networks.

RPL provides a mechanism whereby multipoint-to-point traffic from devices inside the Low-Power and Lossy-Networks (LLNs) towards a central control. It also allows a point-to-multipoint traffic from the central control point to the device inside the LLN [20,21]. RPL involves many concepts that make it a flexible protocol but a complex one [22]. Here are its main properties:

- DODAG (Destination Oriented Directed Acyclic Graph): a topology similar to a tree is adopted in order to optimize routes between sink and other nodes for both the collect and distribute data traffics. Each node within the network has an assigned rank which increases as the nodes move away from the root node. The nodes resend packets using the lowest range as the route selection criteria.
- DIS (DODAG Information Solicitation): is used to solicit a DODAG information object from RPL nodes.
- DIO (DODAG Information Object): is used to construct, maintain the DODAG and to periodically refresh information about nodes and the network topology.
- DAO (DODAG Advertisement Object): is used by nodes to propagate destination information upward along the DODAG in order to update the information of their parents.
- Non-storing mode: RPL routes messages downward using IP source routing.
- Storing mode: RPL routes messages downward by the IPv6 destination address.

### 2.3. Machine Learning Algorithms and Detection Methods

**Figure 2:**
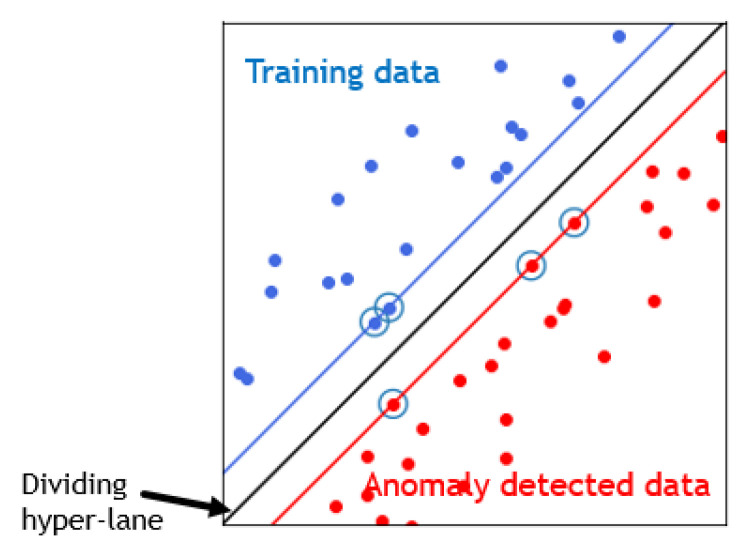
SVM classification.

The proposed anomaly detection system detects abnormal behavior of the system by determining the ordinary behavior and by using it as a baseline. Therefore, any deviation from that baseline is considered as anomaly. Therefore, a machine-learning algorithm SVM (Support Vector Machine) was used to deal with classification problems. Since different parameters are used, the non-linear SVM allows capturing complex relationships between those parameters without having to perform difficult transformation. The idea of SVM is simple: The algorithm creates a line or a hyperplane (Figure 2) [23], which separates data into classes. It uses a mathematical function, named the kernel, to reformulate data. After this transformation, SVM algorithm defines an optimal borderline between the labels. Mainly, it does a set of transformations to find a solution to separate the data based on the labels or outputs defined [24,25].

For the choice of this detection technique, we did rely on the lessons learned from the comparative study in [26] which demonstrates SVM efficiency in WSN (Wireless Sensor Network) anomaly detection in comparison to different machine learning techniques for anomaly detection.

Other existing machine learning algorithms can handle the anomaly detection but this work focus on the evaluation and experimentation of SVM algorithm.

## 3. Related Work

Related works can be classified into four fields: IoT intrusion detection, IoT event detection, IoT e-health systems and Reliable IoT systems. Solutions combining both intrusion detection and event detection are not really developed in the context of IoT systems. Meanwhile, there are many proposed solutions in both topics. Our work has the originality to take the best of IoT solutions in the context of hospital supervision and e-health and to propose an integrated system.

### 3.1. IoT Intrusion Detection Systems

For intrusion detection in RPL-based networks, the methods proposed so far concentrate on different attacks such as rank attacks. In [27], authors present a survey of IDS research efforts for IoT providing different attributes about detection methods, IDS placement strategies and the detection of different kinds of attacks. Authors in [28] provide a specification-based IDS for RPL network as a preliminary study without simulation. A distributed lightweight IDS is proposed in [29]. The IDS considers energy consumption as a parameter where each node in the network monitors its own energy. When energy consumption deviates from the expected value, the IDS considers the node as malicious and eliminates it from the path table in the 6LoWPAN network. SecureRPL (SRPL) proposed in [30] is based on hash-based authentication mechanisms to detect network attacks. However, it is characterized by a high energy consumption compared to solutions based on machine learning algorithms. A specification based IDS is proposed in [31]. The IDS divides the network into small clusters with a number of similar nodes. On each cluster head, an IDS is implemented to monitor the cluster members. A hybrid scheme of anomaly and specification based IDS is proposed in [32] to detect sinkhole and selective forwarding attacks. The proposed ML-based solutions are resource hungry and unsuitable for 6LoWPAN. Authors of [26] compared several unsupervised machine learning approaches based on local outlier factor, near neighbors, Mahalanobis distance and SVMs for intrusion detection. Their experiments show that SVM is the most appropriate technique to detect selective forwarding and jamming attacks. Authors in [33] present an anomaly-based lightweight IDS based on threshold values of RPL messages for detecting attacks on the RPL protocol.

### 3.2. IoT Event Detection Systems

Many research aim to deploy and integrate smart sensors in IoT network. Authors in [34] present a smart house architecture based on IoT. They use multiple sensors based on Zigbee modules which present a short range to use in hospitals. Authors in [35] provide a deep learning algorithm CNN (Convolution Neural Network) for fire disaster to monitor and to identify the abnormality in forest observations. An early-warning safety system is proposed by [36] in Coal mine use-case. Authors present a prototype using sensors for air quality parameters including temperature, humidity and other parameters. For fire detection, authors in [37] designed a wireless sensor network using multiple sensors for early detection of house fires using Global System for Mobile Communications (GSM) as communication system. Authors in [38] propose a prototype for fire detection in outdoor environments based on sensors and Low Power Wide Area Network (LPWAN). Authors focus on the accuracy in the temperature and gas measurements and the real-time detection of fire. In IoT systems for environment observation, the edge computing is hardly used since gateways suffer from energy constraints.

### 3.3. IoT e-Health Systems

Authors in [4] propose an IoT architecture to overcome the limitations of the classical hospital information system. This study presents one of preliminary works for e-health systems. Meanwhile, in other works [3], authors present an e-health gateway, called UT-Gateway which provides efficient local services for health monitoring applications. However, they do not handle network attacks like those in RPL. In [39], authors propose an IoT architecture where collected data from different sensors are processed and analyzed in the cloud. This collection of medical data uses real-time big data analysis. In [40], authors provide a holistic AI-driven IoT e-Health architecture by distributing the intelligence across all levels and comparing ML techniques such as SVM but they do not handle anomaly detection. Authors in [41] present the concept of blockchain as a security mechanism for e-health IoT to enforce the integrity property. Therefore, it should be combined with other security mechanisms including authentication and encryption. In [42], authors introduce an efficient secured group-based lightweight authentication scheme for IoT e-health applications by establishing secure channels between the sensors and a base station using elliptic curve cryptography (ECC) techniques on the group-based nodes.

Compared to these works, the proposed solution targets and addresses anomaly detection at two levels: e-health and networks using a common IoT system. This reduces management costs and improves detection efficiency. The edge-computing and machine learning (SVM) are presented in the proposed solution for efficient detection process.

### 3.4. Reliable IoT Systems

In the context of reliability, a lot of works concentrate on the integrated reliability systems of both software and hardware [43,44]. Subsystem integration allows for the detection of more failures than the subsystems separately. For example, software failures generated by hardware failures and hardware failures caused by software failures are detected in the integrated subsystems and not detected if the subsystems are separately considered [45]. Other works integrate anomaly and failure detection subsystems of multi domains in a single system [46]. The integration allows the optimization of resources and the reduction of management cost. IoT is itself considered as an integrated set of subsystems for observing different targets and different domains. However, management subsystems are still considered as ’silos’ with different targets and tools. In e-health systems, researcher try to enhance the reliability, given the sensitivity of manipulated data and services. For example, an architecture based on network slicing can provide reliability for smart health applications was proposed in [47]. Other studies aim to apply optimization methods as the Multi-Response Optimization (MRO) method for reliability-based performance optimization [48].

## 4. Anomaly Detection Scenarios

In this section, the anomaly detection scenarios are presented in a smart hospital infrastructure. Two scenarios are proposed: one refers to network intrusion detection and the other refers to e-health event detection.

### 4.1. Intrusion Detection Scenario

To ensure the evaluation of the proposed intrusion detection process, The three most common attacks in IoT network were chosen [49,50] to test the proposed solution. These three attacks are described as follows:

#### 4.1.1. Rank Attack Scenario

**Figure 3:**
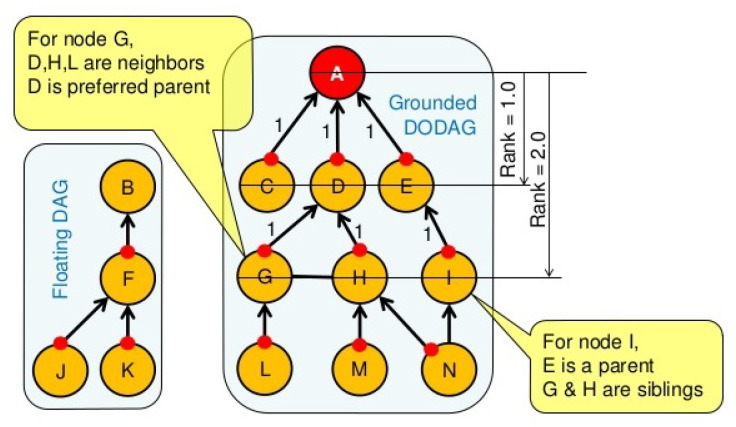
The rank in IoT network.

Rank attack is one of well known attacks against the routing protocol RPL in the network layer of IoT. The rank in RPL protocol, as shown in Figure 3, is the physical position of the node with respect to the border router and neighbor nodes [51].

**Figure 4:**
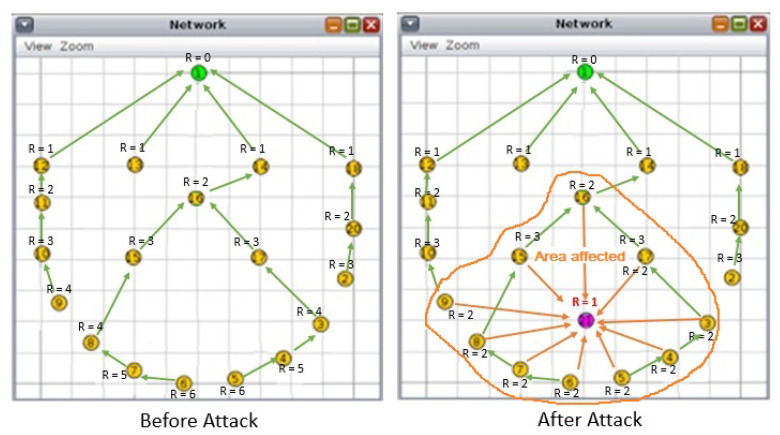
Rank attack.

Since the network is dynamic due to the mobility of its nodes (sensor moving with patients, etc.), the RPL protocol periodically reformulates the DODAG. As shown in Figure 4, an attacker may insert a malicious node into the network to attract other nodes to establish routes through it by advertising false ranks while the reformulation of the DODAG is done [52].

**Figure 5:**
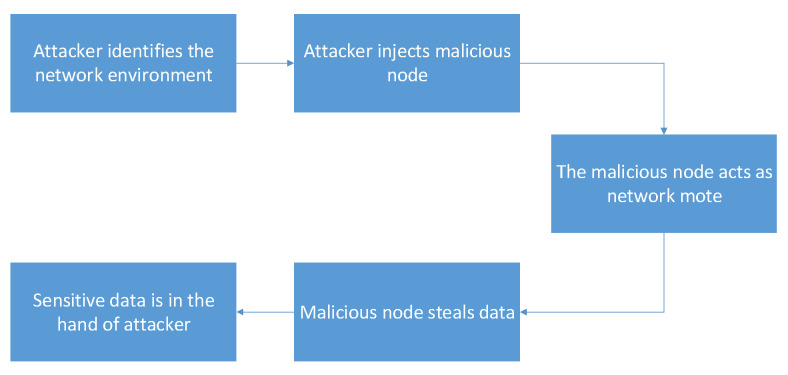
The rank attack scenario.

By default, RPL has the security mechanisms to mitigate the external attacks. However, it can not mitigate the internal attacks efficiently. In that case, the rank attack is considered one of dangerous attacks in dynamic IoT networks since the attacker controls an existing node (being one of the internal attacks that can affect the RPL) in the DODAG. The attacker can also insert his own malicious node, and that node will act as the attack node as shown in Figure 5.

#### 4.1.2. Version Number Modification Attack Scenario

**Figure 6:**
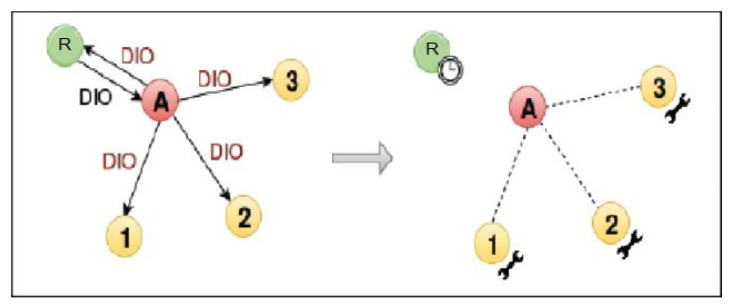
Version number modification attack.

This internal attack is achieved by changing version number (lower to higher) of a DODAG tree. With its modified RPL file, the malicious node increases the version number before forwarding received DIO messages (Figure 6), thus triggering unnecessary global repairs. This increases overhead control and packet loss [53].

#### 4.1.3. Flooding Attack Scenario

**Figure 7:**
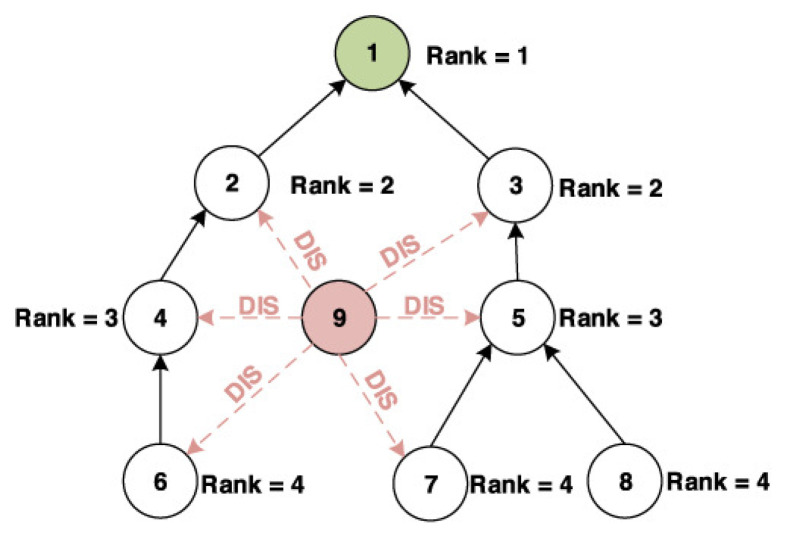
Flooding attack scenario.

Flooding attacks or ICMP flooding, is a DDoS attack where ICMP flood overwhelms the target resource with ICMP Echo Request (ping) packets, generally sending packets as fast as possible without waiting for replies. This type of attack can consume both outgoing and incoming bandwidth, since the victim’s servers will often attempt to respond with ICMP Echo Reply packets, resulting significant overall system slowdown. Thanks to the ContikiRPL configuration constants set with the building block, the malicious node immediately starts sending DIS messages to its neighbors as shown in Figure 7 [54], then triggering DIO messages and trickling timers reset.

### 4.2. Event Detection Scenario

The smart hospital infrastructure is mainly composed of two types of sensors: environmental and body sensors. Environmental sensors are distributed in different rooms as temperature and humidity sensors. Body sensors basically control the human body states such as human body temperature, oxygen level and heart rate sensors. Therefore, to test the event detection process, two scenarios are proposed for each type of sensors described as follows:

#### 4.2.1. Environment Sensors’ Anomaly Scenario

**Figure 8:**
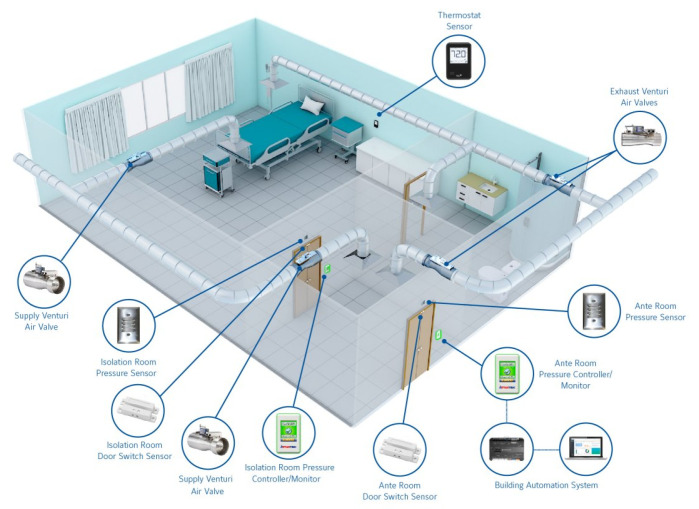
Different environment sensor types.

In smart hospitals, the different rooms are supposed to be equipped with standard environmental sensors as shown in Figure 8 [55]. For example, the operation room should have a low temperature level and normal humidity level. The patient room should be cosy with a modern temperature level. An anomaly can be caused by a high temperature level or a low humidity events that can cause damage to patient health. In these cases, an event detection component is necessary to detect modifications and send alerts whenever an anomaly occurs.

#### 4.2.2. Body Sensors’ Anomaly Scenario

**Figure 9:**
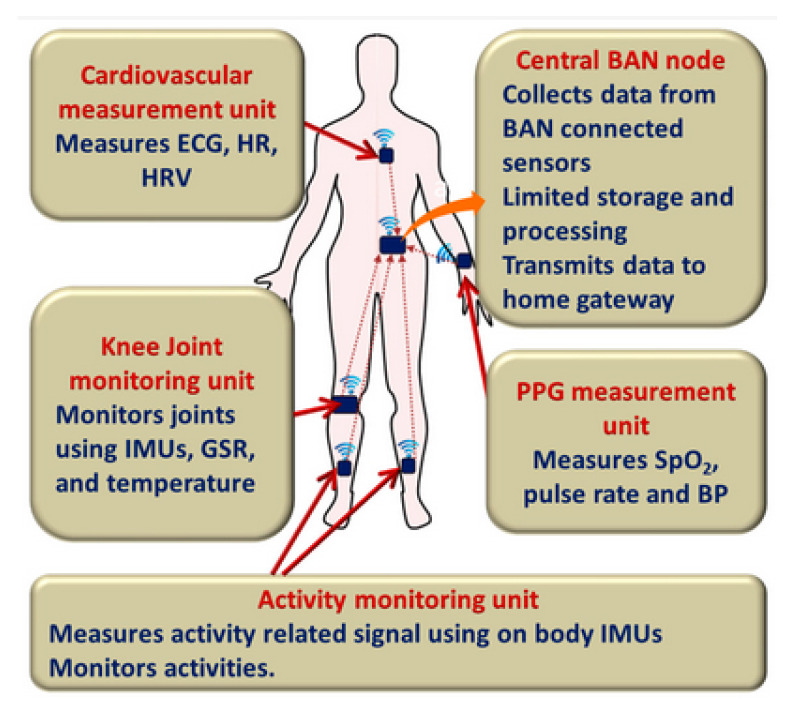
Different body sensor types.

The body sensors are small sensors embedded on the patient body to control his/her health status as oxygen level, heart rate level, body temperature level and other critical parameters that directly affect the human life [56]. Examples are shown in Figure 9.

A body sensor can keep track of the overall health condition of the patient in real-time and provide feedback and support from distant facilities. An event detection component is essential to detect any variation of the human body status.

## 5. Ads for Smart Hospital Iot Systems

This section demonstrates the ADS system architecture and the algorithms used for anomaly detection.

### 5.1. Ads Architecture and Main Components

**Figure 10:**
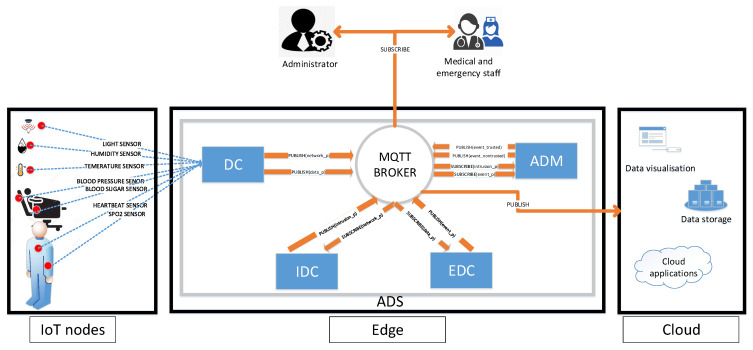
ADS architecture.

The Figure 10 describes the ADS architecture and it’s main component as follows:

#### 5.1.1. Dispatcher Component DC

The DC acts as a packet filter. The received packets from different sensor nodes are treated at DC level. The dispatcher extracts, standardizes and publishes two types of collected data:

- Network data: contains the network parameters’ values and status such as energy consumption and delivery ratio.
- Sensor data: contains sensor related data such as body temperature and heartbeat data.

IDC and EDC receive data related respectively to Network and Sensors.

#### 5.1.2. Intrusion Detection Component IDC

IDC detects anomalies related to the network state. For that, it subscribes to the appropriate topic that allows receiving network related data from the MQTT broker filtered and published by the DC. The received data is classified based on SVM algorithm. Compared to rule-based processing, machine learning algorithms have the ability to detect new forms of attacks in addition to the known ones [57], since a model is build to recognize normal data, and any deviation from such pattern is detected as abnormal data. When an attack is detected, IDC publishes notifications to be received by subscribed users.

#### 5.1.3. Event Detection Component EDC

This component detects anomalies related to environment and patient health states. In fact, the different sensors scattered within the hospital send their data to the border router. EDC subscribes to sensor data topics to receive all data filtered by the DC. EDC analyses received data and publishes detected events such as fires or patient state changes. Like IDC, SVM is used for detecting the events of interest. These events are received in real-time by concerned programs or persons (medical staff or administrators). The main difference between IDC and EDC is the trained information that leads to different models. On the one hand, IDC uses network parameters such as energy and packet delivery ratio. On the other hand, the EDC uses sensing data parameters which are the data collected from different sensors as environmental and body sensors.

#### 5.1.4. Anomaly Decision Manager ADM

The treated data in this context such as data related to human lives is very critical (e.g., if the heartbeat of a patient is very low/high, an immediate medical assistance is required). Therefore, an intrusion can cause data modification and lead to inadequate decisions taken or ignored by the emergency staff (e.g., someone that needs medical assistance, while the sensor sends normal data omitting the need to any medical assistance). For this reason, when the anomaly decision manager receives a notification from IDC, it reacts accordingly depending on the presence or the absence of an attack in the network. When an attack occurs, the ADM adds a tag (non trusted) to the event data alerting the medical staff that the received data may be falsified. When the the network administrator fixes the problem, the system regains back to its normal state. The event data is tagged as trusted. This precaution is taken to avoid risky decisions while the network is under attack. ADM process is described in Algorithm 1.
**Algorithm 1:** Anomaly decision manager algorithm
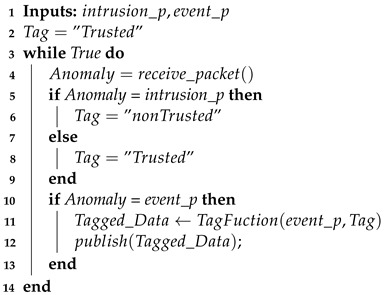


#### 5.1.5. MQTT Broker

MQTT (Message Queuing Telemetry Transport) is a lightweight messaging protocol for IoT applications. Unlike the client/server principle used with HTTP protocol, MQTT uses the one of publication/subscription. Therefore, several component connect to a single server known as MQTT Broker to either publish information or subscribe to it. The MQTT broker plays an important role for the ADS solution linking all system’s components exchanging messages in a loosely coupled way. Therefor, the DC after receiving message from nodes it publish the network parameter (network_p) and the data parameter (data_p). The IDC and EDC component subscribe those parameters and they role to detect the anomaly by publishing intrusion parameter (intrusion_p) for the IDC and event parameter (event_p) for the EDC. And finally, the ADM subscribe if the event is trusted or non-trusted. Indeed, adopting a publish/subscribe broker allows the independence between sensors and the applications processing the collected data. It is possible to add sensors to the system without modifying the applications. The opposite is also true, subscribers to events can be added or removed without any impact on the sensors side.

### 5.2. ADS Placement Choice and Integration

One of the important decision in anomaly detection is the placement of the ADS in the network. Since data is critical and the latency of applications is sensitive. A centralized approach is chosen by installing the ADS at the edge of the network (border router). Therefore, it can analyze all the packets that pass through it. The choice of the centralized ADS is generally adopted to avoid the placement of ADS modules in constrained devices [58]. However, energy issue is not an issue for the border router installed in a hospital. In opposition, the nodes as body sensors are constrained devices and may be alimented with batteries. The choice of the edge placement rather than the cloud placement reduces the communication overhead when data is sent from the hospital to the cloud. Indeed, when an anomaly occurs, there is no need to send non-trusted or faulty data. Instead, the ADS logs locally all information about the anomaly and only alarms are reported remotely [27,58,59].

The proposed ADS is designed to be integrated easily in existing e-health IoT systems. E-health system managers and network administrators should define properly the anomalies related to patients, environment or network intrusions to be detected. After that, a feature extraction phase should be applied to identify relevant features to built specific models for anomaly detection.

## 6. Evaluation and Results

In this section, four major parts are presented:

- The first part deals with the proposed architecture where a real prototype is implemented from sensors to the cloud as a proof of concepts.
- The second part deals with the adopted simulation process to implement different attacks with multiple nodes.
- The third part describes data-sets used for the proposed solution.
- The last part presents the evaluation of the proposed system.

### 6.1. Experimentation Setup

**Figure 11:**
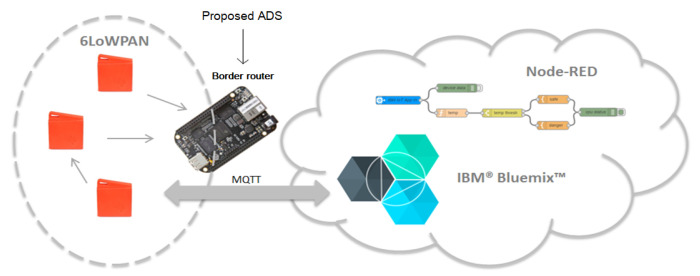
System architecture.

A prototype of the proposed solution is implemented as a proof of concepts as shown in Figure 11. The prototype consists of the following elements:

- TI SensorTag CC2650: this low power consuming sensor node measures the variation of physical parameters including light, magnetic sensor, humidity, pressure, acceleration and ambient temperature [60]. Two sensing nodes are used in the prototype.
- Border router: A BeagleBone Black (BBB) equipped with CC2531 USB Dongle as RF interface is used. Linux Debian is used as an operating system. Appendix A details the border router configuration.
- Cloud platform: are computer system resources that provide a series of modular cloud services including computing, data storage, data analytic and machine learning tools.
- IBM Watson IoT Platform: this platform allows to connect any IoT nodes or gateway to start sending data securely up to the cloud using the open, lightweight MQTT messaging protocol. It allows to set up and manage devices using a dashboard and secure APIs, so that applications can access and use live and historical data.
- Node-RED: a programming tool for wiring together hardware devices, APIs and online services.

The architecture of the system presented in Figure 11 displays the TI sensors sending data to the border router for analysis. The collected sensor data is visualized through IBM Watson IoT Platform (component of IBM Bluemix). Then, data may be treated with NODE RED for advanced analysis.

Python programming language is used to implement the proposed ADS components and run them over the border router as python scripts. Mosquitto was considered as the MQTT broker.

After deploying the current prototype, it shows an efficiency in terms of collecting sensor data in real-time and sending information to the border router for analysis and then to the cloud for visualization. The event detection process is achieved and tested using this prototype with two different datasets. However, it would not be possible to test real RPL attacks. Therefore, a simulation process is required using a larger number of nodes.

### 6.2. Simulation Setup

**Figure 12:**
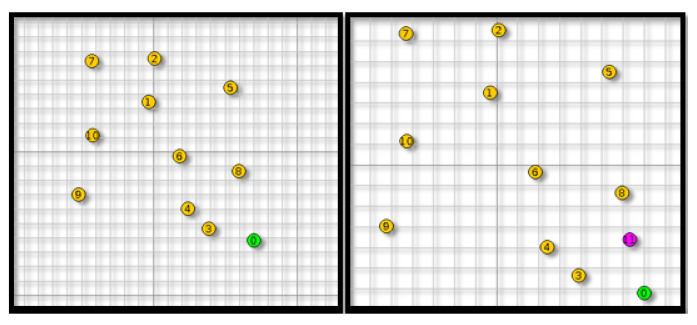
Simulation topology.

To investigate the effectiveness of the proposed ADS, an anomaly scenario is implemented using Contiki-Cooja simulator [61]. This part presents the simulation setup and evaluation metrics. In the Contiki Cooja simulator nodes are referred as motes, and they have the same meaning. A benchmark containing 11 motes spread across an area of 200×200 s (Simulation of an area of a hospital where different sensors are placed in different locations to control the patient rooms or embedded on patients bodies to collect their health status data) is used. The collected sensor node data for both environmental and body were injected in the Contiki Cooja node. There is one sink (mote ID:0 with green dot) and 10 senders (yellow motes from ID:1 to ID:10). Every mote sends a packet to the sink at the rate of 1 packet every 1 minute. The centralized ADS is implemented at the root mote (the sink) in order to collect and analyze network data. Malicious mote (purple color) is introduced in a random position, as shown in Figure 12. Table 1 summarizes the used simulation parameters. Afterwards, both of anomaly detection components (IDC and EDC) are analyzed. The simulation scenarios are run for one hour with and without attacks with two kinds of topology.

- Topology 1: IoT network without malicious motes.
- Topology 2: IoT network with one randomly placed malicious mote.

The accuracy of the proposed IDC is evaluated based on energy consumption features. Power tracking data are collected per mote in terms of radio ON energy, radio transmission TX energy, radio reception RX energy, and radio interfered INT energy. In order to calculate energy consumption, the Equations (1) and (2) are used [62] based on parameters in Table 2, as follows:

(1) Energy(mJ)=(transmit×19.5mA+listen×21.8mA+CPU×1.8mA+LPM×0.0545mA)×3V/4096×8

(2) $$Power\;\left(\mathrm{mW}\right)=\frac{Energy\;(\mathrm{mJ})}{Time\;(s)}$$

**Figure 13:**
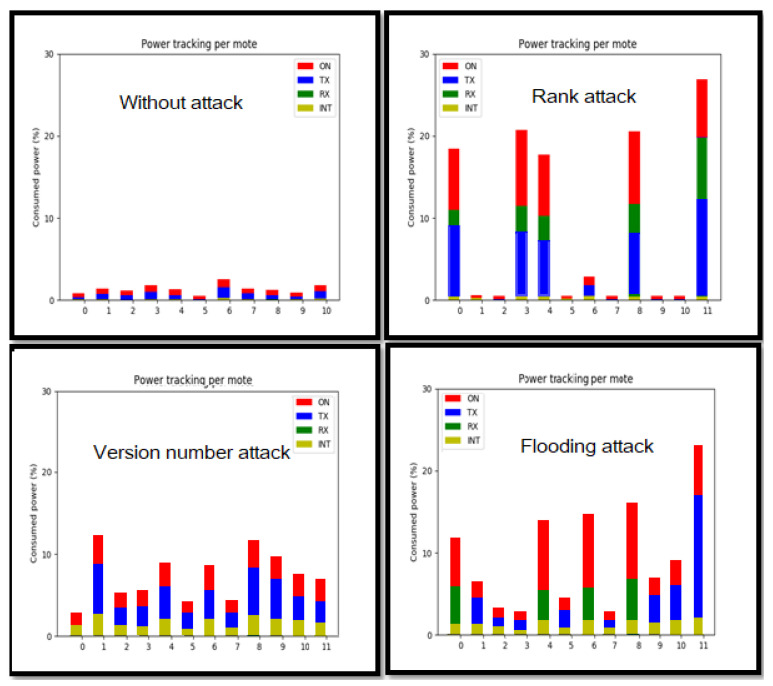
Power tracking per each mote for each attack.

Data containing 1000 instances of consumed energy values are used for each node in the network. Figure 13 presents the evolution of power tracking of each node in the four different scenarios (one scenario without attacks and remaining scenarios with the three previously described attacks):

- Scenario 1: in this scenario, a network without attacks is simulated, and energy consumption is collected. Two colors are the overwhelming colors in the graph. The red color indicates the energy while the node is active, and the blue color indicates the energy while the node is sending. The two other colors are less present. This means that the node does not consume much energy while receiving data, and the network does not present much interference. All of the sensor nodes show a regular energy consumption in terms of receiving (node 0) and sending energy (nodes from 1 to 10). The purpose of this simulation is to collect training data for the proposed IDC.
- Scenario 2: in the rank attack scenario, the collected data exhibits five motes with high level of energy consumption in comparison to the rest of motes:
  - The malicious mote 11 sends false rank messages, and the other motes send DIO messages to reconstruct the DODAG tree. The malicious mote sends back; therefore, the energy consumption in terms of sending (TX), receiving (RX), and power (ON) is very high.
  - Motes 0, 3, 4, and 8 that are close to the malicious mote show high energy consumption in terms of sending (TX), receiving (RX), and power (ON). This is explained by the fact that, while the malicious mote (mote 11) is sending DIO messages, the nearby nodes are receiving them and sending back responses.
- Scenario 3: in the scenario of version number attack, all of the motes in the network act uncommonly:
  - The malicious mote 11 sends messages to the nearest motes by increasing the version number before forwarding received DIO messages, thus triggering unnecessary global repairs.
  - The nearby motes are the first affected motes by the malicious mote by trying to repair the DODAG in the network. Therefore, they send back those repairing messages to their neighbors, which overwhelms the network.
- Scenario 4: in the scenario of Flooding attack, after the observation of the collected data. Different variations of energy consumption are obtained:
  - The malicious mote 11 sends many messages to the other nodes. Therefore, a high (TX) is observed.
  - The nearby motes 0, 4, 5, and 8 are the most affected by the flooding attack. Therefore, they present a high (RX).
  - The other motes act normally, because the near motes will not forward the received messages from the malicious mote. Therefore, the flooding attack only affects the surrounding area.

Figure 13 shows energy consumption variation in different scenarios. These collected data are used for ADS evaluation.

### 6.3. Data-Sets

Three data-sets were used to test the efficiency of the proposed solutions.

#### 6.3.1. Idc Data-Set

In the first scenario, while using simulation, IDC data-set is collected for training. This data-set is relative to the normal behavior of the network. More precisely, the consumed energy is collected in terms of radio energy (ON), radio transmission (TX) energy, radio reception (RX) energy, and radio interfered (INT) energy. 1000 instances are used for training and 200 instances for test. For testing the algorithm, three attack scenarios are simulated.

#### 6.3.2. EDC Data-Sets

In order to evaluate the efficiency of EDC, a data-set containing 1000 instances of environmental data composed of temperature, humidity, and light [63] is used for training the SVM algorithm, while 200 instances are used for test. For testing the algorithm, a scenario of fire is simulated by increasing the temperature and light data values and decreasing the humidity data values. For the body sensor data, a data-set containing 1000 instances of human body data [64] is used for training the proposed algorithm. Data are composed of heart rate and body temperature information. 200 instances are used for testing. For testing the algorithm, the scenario of heart attack is simulated by decreasing heart rate data.

### 6.4. Evaluation of Detection Accuracy

In this work, the performance of the proposed system was evaluated based on the Anomaly Detection Rate (ADR) metric, which identifies the rate of abnormal events and observations in a period of time [57]. This metric is considered to be one of most important metrics, since the main problem in wireless networks is the integrity of exchanged data. In this context, ADR allows for assessing the accuracy of detection of anomalies that are related to data or networks. ADR is defined in the following Equation (3).

(3) $$ADR=\frac{\mathrm{Abnormal}\;\mathrm{events}\;\mathrm{detected}\;\mathrm{by}\;\mathrm{SVM}}{\mathrm{Total}\;\mathrm{data}\;\mathrm{amount}}$$

Table 3 and Table 4 show the results of simulations for network attacks.

IDC detects two types of routing attacks with high ADR (Rank attack and Version number modification attack are detected with an ADR higher than 90%), as shown in Table 3. Flooding attack is detected with a low ADR. This may be explained by the countermeasures used in the simulator to regain its normal state after the flooding attack is done.

The level of temperature is increased and the level of humidity is decreased in order to simulate an abnormal behavior such as a fire. EDC detects the environment change with a high ADR. Additionally, for the heart attack scenario, the EDC detects abnormal body status with a high ADR. Table 4 shows the results.

A one-class SVM with non-linear kernel (RBF) with parameters (nu = 0.1, kernel = “rbf” and gamma = 0.1) [65] is implemented in both EDC and IDC. As a programming language, Python v3.6 is used, and the machine learning library scikit-learn is considered to implement One-class SVM.

The proposed ADS in Table 3 and Table 4 presents accurate detection rates for different scenarios with different types of anomalies (network intrusion, fire, and a heart attack).

## 7. Conclusions

Reliability in IoT is a vital topic, since e-health data have to be exchanged efficiently. In this paper, an Anomaly Detection System “ADS” is proposed for smart hospital infrastructures with two modules: IDC for detecting network anomalies and attacks, and EDC to detect e-health related events. Both of the modules IDC and EDC interact efficiently in a common and unified system, which leads to an ease of administration and an optimization for system management cost. As soon as IDC reports an intrusion, the events that are detected by EDC are no longer trusted and are carefully considered. Such reliability is very important in hospital infrastructures to avoid sending false alerts or taking incorrect decisions about patients’ health care. The ADS placement strategy is fully centralized and performed at the edge router. The choice of edge computing is motivated by the sensitiveness of data that may be intercepted or altered if sent to the cloud. Furthermore, processing data close to the sources significantly reduce the decision latency and network bandwidth. This work has the originality to integrate the two anomaly detection systems (e-health monitoring and infrastructure supervision) in a single system. This has the advantage of improving the overall system reliability and, then, providing accurate e-health decision making. Furthermore, subsystems’ integration optimizes resource management.

For future work, the following topics will be investigated:

- Model optimization and best parameters tuning as well as comparison with other machine learning algorithms.
- Developing models for detecting other specific internal attacks, such as local repair attack.
- Considering the mobility of nodes in detecting attacks.
- Comparing different machine learning techniques and using online learning.
- Using multiple parameters as: packet delivery ratio, average time, and latency.

## Figures and Tables

**Table 1:** Simulation parameters.

Parameter	Value
Platform	Cooja Contiki 3.0
Number of nodes	10 senders, 1 sink
Topology	star
Area	200 m × 200 m
Sending rate	1 packet/minute
Simulation run time	1 h
Radio Medium Model	Unit Disk Graph Medium (UDGM): Distance Loss
Mote Type	Tmote Sky
Duty Cycle	ContikiMAC
Range of Nodes	Rx and Tx: 50 m, Interference: 100 m
Physical Layer	IEEE 802.15.4
MAC Layer	ContikiMAC
MAC Driver	CSMA/CA
Channel Selection	By Default 26
Network Layer	ContikiRPL
Transport Layer	UDP
Traffic model	Constant Bit Rate

**Table 2:** Equation parameters description.

Variables	Meaning
LPM	Power consumption parameter that indicates the power used when in sleep condition
CPU	Power parameter that indicates the level of node processing
Transmit	Parameter related to node communication while transmitting
Listen	Parameter related to node communication while receiving

**Table 3:** Anomaly detection rates for different attacks.

Anomaly (IDC Detection)	ADR (%)
Rank attack	93.4
Flooding attack	60.8
Version number modification attack	91.6

**Table 4:** Anomaly detection for event scenario.

Anomaly (EDC Detection)	ADR (%)
Event ( Increasing temperature level)	85.7
Event ( Heart attack)	82

**Figure A1:**
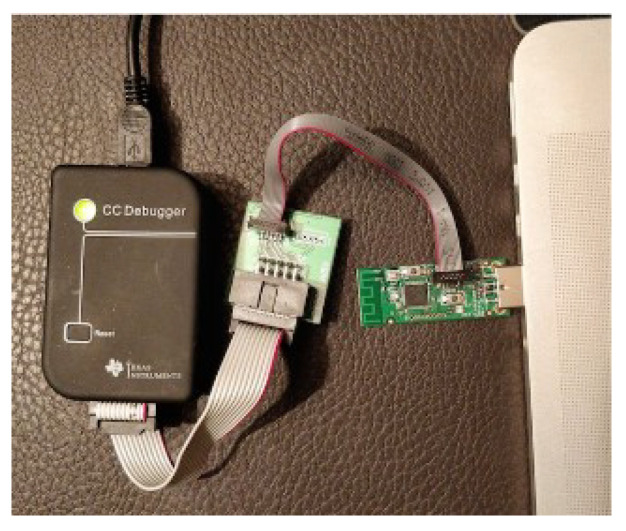
CC debugger + USB dongle.

**Figure A2:**
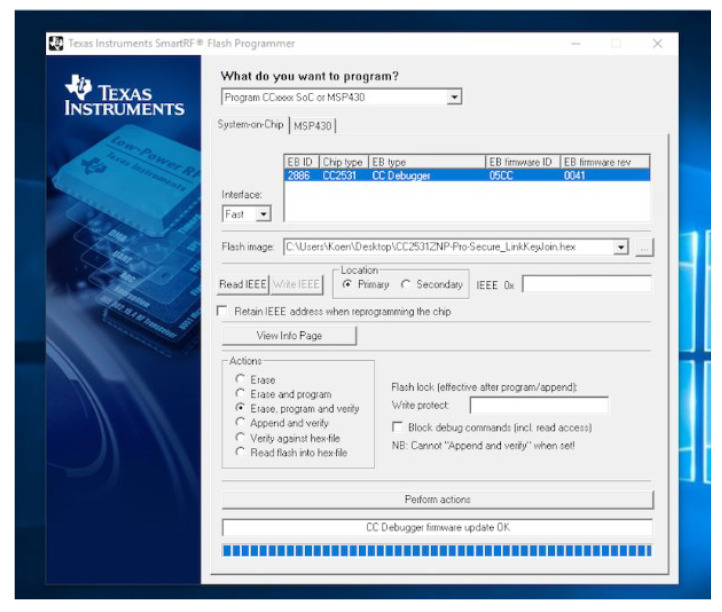
Patching the USB dongle through SmartRF Flash Programmer.

**Figure A3:**
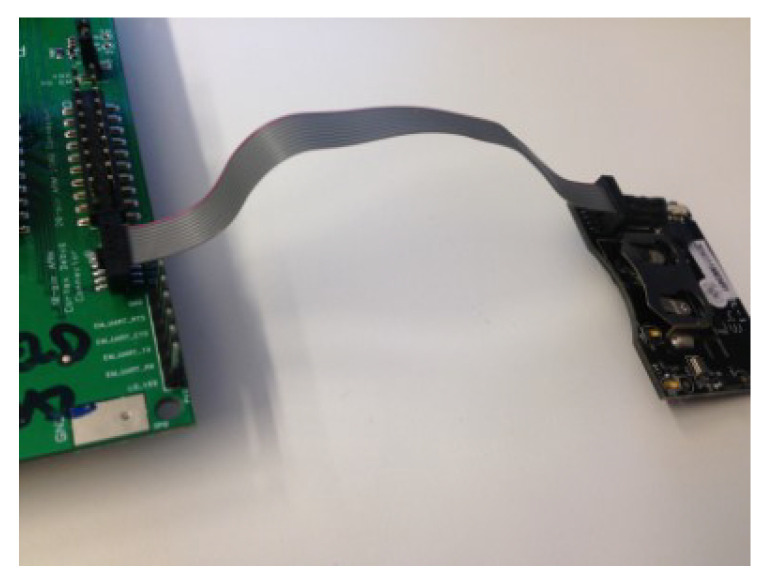
SmartRF06 evaluation board + TI CC2650 SensorTag.

**Figure A4:**
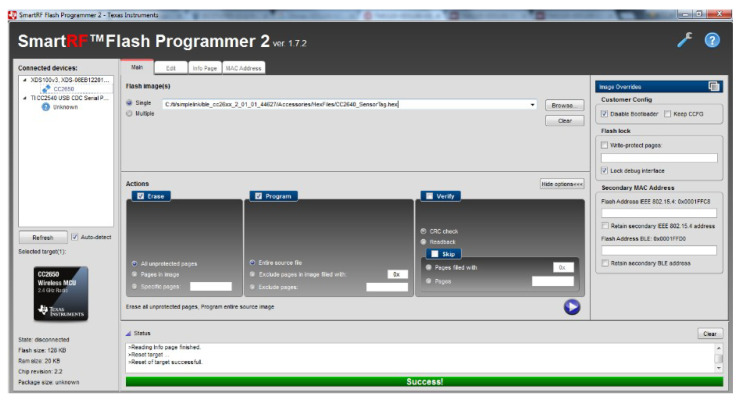
Patching the SensorTag through SmartRF Flash Programmer v2.

**Figure A5:**
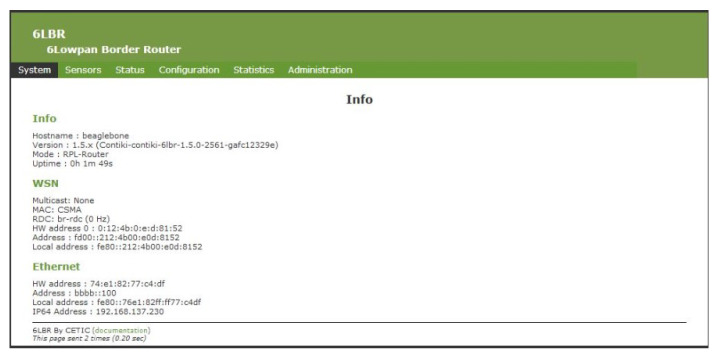
BeagleBone Black: Home page.

**Figure A6:**
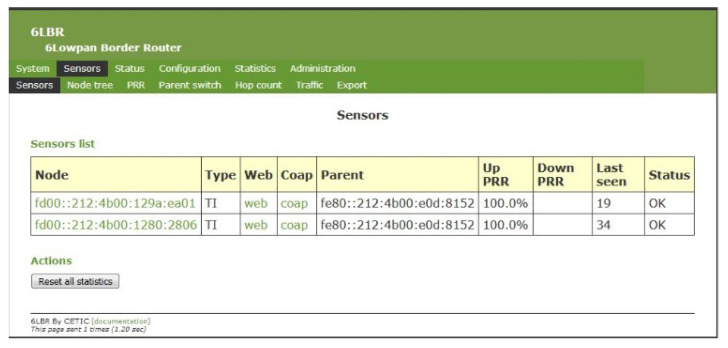
BeagleBone Black: Sensors page.

## Data Availability

Not applicable.

## Abbreviations

The following abbreviations are used in this manuscript:

6LoWPAN	IPv6 over Low Power Wireless Private Area Network
ADR	Anomaly Detection Rate
ADS	Anomaly Detection System
API	Application Programming Interface
BBB	BeagleBone Black
DC	Dispatcher Component
DDoS	Distributed Denial of Service
EDC	Event Detection Component
ICMP	Internet Control Message Protocol
IDC	Intrusion Detection Component
MQTT	Message Queuing Telemetry Transport
RBF	Radial Basis Function
RPL	Routing Protocol for Low-Power and Lossy Networks
SVM	Support Vector Machines
WSN	Wireless Sensor Network

## Appendix A. Border Router Configuration

The BeagleBone Black running Debian Linux was equipped with CC2531 USB Dongle (2.4 GHz) as RF interface. 6LBR (6LoWPAN/RPL Border Router solution) that is a deployment-ready platform for interconnecting IP and 6LoWPAN networks is used to implement the border router application. It assumes an Ethernet interface on the IP side and an 802.15.4 interface on the 6LoWPAN side. Sensor tags act as 6LoWPAN nodes.

The list of used hardware and software is presented in the following:

- **Hardware list:**
  - BeableBone Black.
  - Micro SD card (4GB).
  - Network Cable (RJ-45).
  - Mini-USB[M] to Standard-USB[M] cable.
  - SmartRF06 evaluation board.
  - CC debugger.
  - TI CC2650 Sensor Tag.
- **Software list:**
  - SmartRF Flash programmer.
  - SmartRF Flash programmer v2.
  To configure the BBB, the following steps are used:
  1. Download the latest BBB Debian software image.
  2. Patch the BBB using Balena Etcher.
  3. Patch the USB Dongle with the appropriate firmware using the CC debugger and SmartRF Flash Programmer.
  4. Install the 6LBR application on the BBB allowing communication between both networks.
  5. Configure the PAN-ID into 0xABCD and the channel into 25.
  6. Install Wrapsix NAT64 service in order to connect a 6LoWPAN devices to the Internet.
  7. Patch the mesh devices with the appropriate firmware using the SmartRF06 evaluation board and SmartRF Flash Programmer v2.
  8. Run 6LBR application and open navigator and tap the 6LBR address.
  9. After the sensors are connected to the Internet, we can consult the sensor data in the IBM Waston IoT platform using the sensor ID.
